# Basolateral and central amygdala differentially recruit and maintain dorsolateral striatum-dependent cocaine-seeking habits

**DOI:** 10.1038/ncomms10088

**Published:** 2015-12-14

**Authors:** Jennifer E. Murray, Aude Belin-Rauscent, Marine Simon, Chiara Giuliano, Marianne Benoit-Marand, Barry J. Everitt, David Belin

**Affiliations:** 1 Department of Psychology, University of Cambridge, Cambridge CB2 3EB, UK; 2 Behavioural and Clinical Neuroscience Institute of the University of Cambridge, Cambridge CB2 1QB, UK; 3 Department of Pharmacology, University of Cambridge, Cambridge CB2 1PD, UK; 4 Groupe de recherche en psychiatrie, Paris GDR3557, France; 5 Laboratoire de Neurosciences Expérimentales et Clinique, INSERM, U1084, Poitiers F-86022, France; 6 Laboratoire de Neurosciences Expérimentales et Cliniques, Université de Poitiers, Poitiers F-86022, France

## Abstract

In the development of addiction, drug seeking becomes habitual and controlled by drug-associated cues, and the neural locus of control over behaviour shifts from the ventral to the dorsolateral striatum. The neural mechanisms underlying this functional transition from recreational drug use to drug-seeking habits are unknown. Here we combined functional disconnections and electrophysiological recordings of the amygdalo-striatal networks in rats trained to seek cocaine to demonstrate that functional shifts within the striatum are driven by transitions from the basolateral (BLA) to the central (CeN) amygdala. Thus, while the recruitment of dorsolateral striatum dopamine-dependent control over cocaine seeking is triggered by the BLA, its long-term maintenance depends instead on the CeN. These data demonstrate that limbic cortical areas both tune the function of cognitive territories of the striatum and thereby underpin maladaptive cocaine-seeking habits.

## Abstract (web summary)

Drug seeking behaviour has habitual neural substrates. Here, Murray *et al*. show that the basolateral amygdala and central nucleus of the amygdala are necessary to recruit and maintain, respectively, the intrastriatal functional transition that underlies cocaine seeking habits.

## Graphical abstract

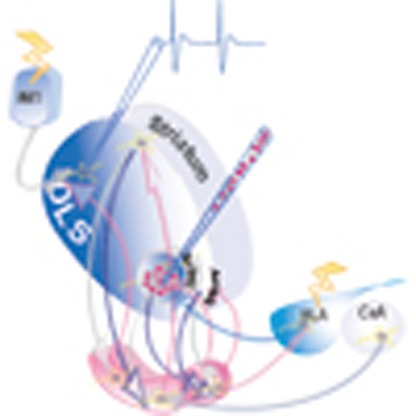

Cocaine addiction is increasingly considered to stem from progressive neural adaptations to chronic drug exposure^1^,^2^,^3^ subserving a loss of control over drug-seeking behaviour that has become established as habitual^4^ and predominantly controlled by drug-associated conditioned stimuli in the environment^5^. However, the mechanisms whereby maladaptive cocaine-seeking habits are established are not yet understood.

The initiation of cocaine seeking as a goal-directed behaviour^6^,^7^ depends on limbic cortico-striatal networks including the nucleus accumbens (Acb) and its functional interactions with the basolateral amygdala (BLA)^8^. While the core of the Acb (AcbC) is suggested to translate drug-related motivation into drug-seeking actions^4^,^9^, the BLA encodes Pavlovian incentive associations^9^,^10^ and through its projections to the AcbC^11^ underlies the performance of prolonged sequences of drug seeking reinforced by drug-associated conditioned stimuli (CS) acting as conditioned reinforcers^12^, bridging delays to drug taking^4^. Thus, the BLA and its interactions with the AcbC are necessary for the acquisition of voluntary cue-controlled cocaine-seeking behaviour.

However, cocaine addiction is not simply a problem of voluntary, or ‘recreational', drug use, but is instead characterized by a compulsive drug-seeking habit^4^, the transition to which has been shown neurally to depend on functional shifts in the control over behaviour from the ventral to the anterior dorsolateral striatum (aDLS)^5^,^13^,^14^,^15^,^16^. Thus protracted exposure to cocaine recruits neurobiological adaptations in the striatum that progressively spread from the AcbC to the aDLS^14^,^17^,^18^,^19^,^20^ finally to consolidate aDLS^6^,^21^, dopamine-dependent cocaine-seeking habits^13^,^21^ and compulsivity^22^,^23^, a hallmark of addiction^24^.

This intrastriatal functional shift has been suggested to depend on the recruitment of the ascending striato-nigro-striatal spiralling dopamine circuitry^25^,^26^, which allows control by the AcbC over dorsal striatal dopaminergic processes^5^,^13^,^27^, thereby linking aberrant drug-related incentive motivational processes to a more rigid habit system^5^. However, the neural mechanisms that underlie this striatal plasticity in the control over drug seeking are poorly understood.

Despite having no direct projections to the aDLS^28^, as opposed to the posterior dorsomedial striatum^28^, the BLA is both a key determinant of AcbC activity^11^,^29^, which is in turn necessary to recruit DLS-dopamine-dependent cocaine-seeking habits^13^, and also of the recruitment of aDLS-dependent functions such as response learning in a spatial navigation task^30^,^31^. In addition, synaptic plasticity at the BLA–AcbC pathway is involved in both appetitive learning^32^, reward seeking^29^ and the strengthening of drug conditioned reinforcement (incubation of cocaine ‘craving') in rats^33^, that may presage the association between cue-evoked cocaine craving and increased dopamine transmission in the aDLS observed in humans^34^. We therefore hypothesized that the BLA, through its functional interactions with the ventral striatum, is the neural locus that triggers the recruitment of DLS-dependent cocaine-seeking habits.

Behaviourally, habitual cocaine seeking has been operationalized as instrumental responding in over-trained second-order schedules of reinforcement^35^,^36^ (SOCR) procedures^5^,^15^,^37^,^38^ whereby drug seeking during prolonged time periods is under the direct control of the conditioned reinforcing properties of cocaine CSs^39^.

We therefore tested the hypothesis that functionally disconnecting the BLA from dopaminergic transmission in the aDLS may prevent the establishment of cue-controlled cocaine-seeking habits. Thus, we combined a pretraining unilateral excitotoxic lesion of the BLA (see Supplementary Fig. 1 for the timeline of the experiments), which we predicted would prevent the cocaine-induced functional recruitment of aDLS-dopamine-dependent control over cocaine seeking on this side of the brain, with dopamine receptor blockade in the contralateral aDLS. Such an asymmetric manipulation bilaterally disrupts—or disconnects—the functional circuitry linking the BLA to dopaminergic transmission in the DLS recruited via the AcbC^13^.

As predicted, we show that the BLA, which controls the activity of the aDLS through AcbC glutamatergic mechanisms, is required to recruit aDLS control over cocaine seeking. We further demonstrate that a functional shift occurs in the amygdala from the BLA to the central nucleus (CeN) over the course of cocaine seeking such that it is the CeN, and not the BLA, that maintains aDLS-dopamine-dependent control over behaviour when cocaine seeking has become habitual.

## Results

### BLA recruits aDLS control of cocaine-seeking habits

**Figure 1:**
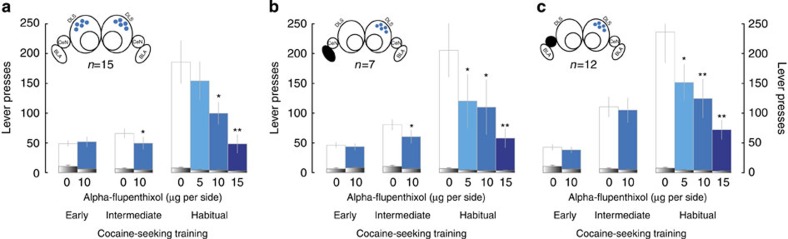
Basolateral and central amygdala are both necessary to recruit dorsolateral striatum, dopamine-dependent, cocaine-seeking habits. Early-stage cocaine seeking was sensitive neither to bilateral aDLS infusion of α-flupenthixol (10 μg per side^13^,^40^,^41^) (*n*=15) (**a**, left panel) nor to BLA–aDLS (*n*=7) or CeN–aDLS (*n*=12) disconnections (**b**,**c**; left panel). Active lever responses in each group were impervious to these manipulations (main effect of group, dose, group × dose or lever × dose interaction: Fs_1,20_<1). When rats had an intermediate training history in cocaine seeking (**a**,**b**; middle panel) their responses increased significantly (lever × time interaction: F_1,21_=13.76 *P*<0.01, partial *η*^2^=0.39) and became slightly sensitive to both bilateral aDLS-dopamine receptor blockade (**a**) or BLA–aDLS disconnections (**b**) (main effect of group: F_1,20_=1.17, *P*=0.29; dose: F_1,20_=9.98, *P*<0.01, partial *η*^2^=0.33), selectively on active lever presses (lever × dose interaction: F_1,20_=4.59, *P*<0.01, partial *η*^2^=0.18). The CeN was not involved in this initial recruitment of aDLS-dopamine-dependent control over behaviour (main effect of lever: F_1,11_=31.59, *P*<0.001 partial *η*^2^=0.39; dose and dose × lever interaction: Fs<1) (**c**, middle panel). However, at a later stage of training, when cocaine seeking has become habitual, while BLA–aDLS-dopamine-dependent disconnection and bilateral aDLS-dopamine receptor blockade further dose-dependently reduced active lever presses (**a**,**b**; right panel) (46% decrease at the unit dose of 10 μg per side and up to 73% at 15 μg) (main effect of group: F_1,20_<1, lever: F_1,20_=56.69, *P*<0.01 partial *η*^2^=0.73; dose: F_3,60_=8.52, *P*<0.01, partial *η*^2^=0.29 and dose × lever interaction: F_3,60_=7.93, *P*<0.01, partial *η*^2^=0.28), CeN–aDLS disconnection resulted also in a dose-dependent decrease in active lever presses (main effect of dose: F_3,33_=4.24, *P*<0.05 partial *η*^2^=0.28 and dose × lever interaction: F_3,33_=4.24, *P*<0.05, partial *η*^2^=0.26). Inactive lever presses are represented in black. *P<0.05, **P<0.01 versus vehicle, Dunnet *post hoc* test.

Permanent pretraining unilateral BLA lesions (see Supplementary Fig. 2 for histological assessment) did not impair the acquisition of cocaine self-administration nor the acquisition of cue-controlled cocaine-seeking behaviour under a SOCR (Supplementary Fig. 3A). Goal-directed^6^, cue-controlled cocaine seeking, measured at an early stage of training (Supplementary Fig. 1), was sensitive neither to bilateral aDLS-dopamine receptor blockade nor to the BLA–aDLS disconnection manipulation (Fig. 1a,b left panel). Thus disconnecting the BLA from aDLS-dopamine transmission does not impair cue-controlled cocaine seeking when we have previously shown it to be goal-directed and dependent on the AcbC and the posterior dorsomedial striatum^40^, but not the aDLS. However, when rats had an intermediate history of training in cocaine seeking, associated with a moderate increase in active lever presses (from 47 to 73 active lever presses in 15 min) (Fig. 1a,b middle panel), behavioural control over cocaine seeking began to devolve to the aDLS as revealed by a limited (27%) but significative effect on cocaine seeking of both bilateral aDLS-dopamine receptor blockade or BLA–aDLS disconnection.

This initial functional recruitment of the aDLS becomes progressively more established over several weeks of training, as shown in our previous work^40^,^41^, reflecting the emergence of a DLS-dependent cocaine-seeking habit under the control of cocaine-associated CSs^5^. Thus, as previously described^13^,^21^,^40^, at this later stage of training, cocaine seeking was dose-dependently decreased by bilateral aDLS infusions of the non-selective dopamine receptor antagonist α-flupenthixol (Fig. 1a right panel), an effect that was dependent on both D1 and D2 dopamine receptors as it was also observed after aDLS infusions of SCH23390 or raclopride, selective antagonists of D1 and D2 receptors, respectively (Supplementary Fig. 4). Moreover, cocaine seeking was similarly dose-dependently decreased by disconnecting the BLA from aDLS dopaminergic mechanisms (Fig. 1b right panel). Thus a pretraining unilateral BLA lesion prevented the functional recruitment of the aDLS ipsilaterally, such that otherwise ineffective unilateral dopamine receptor blockade in the contralateral aDLS now resulted in a decrease in cocaine seeking to the same extent as that seen following bilateral aDLS manipulations in non lesioned rats.

The effect of causal manipulations of the amygdalo-striatal circuitry on cocaine seeking was attributable to a functional disconnection between the BLA and the DLS because neither unilateral DLS dopamine receptor blockade, nor a double ipsilateral manipulation, impaired instrumental performance (Supplementary Fig. 5). Thus, the BLA is required to recruit intrastriatal functional shifts subserving the development of cocaine-seeking habits.

### CeN maintains aDLS control over cocaine-seeking habits

Recent evidence that appetitive memories are associated with long-term potentiation of the BLA–AcbC pathway and concomitant long-term depression of the BLA–CeN pathway^32^ suggests that BLA control over striatal mechanisms as observed here is functionally independent of its connections with, and circuitry involving, the CeN, and thus does not rely on serial processing (BLA-to-CeN) in the amygdala. However, the BLA and CeN have also been shown to be involved in parallel and independent processes^42^; for example, the CeN is functionally linked to the aDLS through its direct projections to the substantia nigra dopaminergic neurons^43^,^44^, and via this pathway mediates the invigoration of instrumental responding in the presence of conditioned Pavlovian excitors^45^,^46^ and also biases motivation towards specific rewards^47^. Thus the CeN projections to the substantia nigra may also be involved in intrastriatal functional shifts. This alternative amygdalo-striatal route, shown to be involved in habitual control over behaviour for natural reinforcers^48^, may serve as a regulatory route, transmitting arousal caused by presentation of cocaine CSs^49^ to the aDLS-dependent habit system. We therefore investigated whether disconnecting the CeN from the aDLS could also influence the recruitment of a aDLS-dependent cocaine-seeking habit.

As predicted, when cocaine seeking did not depend on the aDLS, that is, early after training^40^,^41^, a disconnection combining a selective lesion of the CeN with contralateral aDLS-dopamine receptor blockade had no effect on cocaine seeking (Fig. 1c left panel). In marked contrast to the effects of a bilateral aDLS manipulation or the BLA–aDLS disconnection, the CeN–DLS disconnection also had no effect on cocaine seeking after intermediate training (Fig. 1c middle panel). However, in contrast, this disconnection markedly decreased well-established, habitual, seeking responses by 70% (Fig. 1c right panel). The influence of the asymmetric manipulation of the CeN and dopaminergic transmission in the aDLS is attributable to a functional disconnection, since a unilateral CeN lesion or a double ipsilateral manipulation had no effect on the acquisition of cocaine self-administration, cocaine seeking (Supplementary Fig. 3) or the expression of cocaine-seeking habits (Supplementary Fig. 5C). Thus the CeN is involved in the long-term maintenance of cocaine-seeking habits by the aDLS.

The lack of effect of the CeN–aDLS disconnection at an intermediate stage of training suggests that the CeN is functionally recruited later to exert an influence on the aDLS control over cocaine-seeking behaviour, consolidating a process initiated by the BLA.

We therefore sought further to investigate whether the BLA and the CeN were indeed controlling successive, non-overlapping, stages of intrastriatal, dopamine-dependent, functional shifts subserving cocaine-seeking responses^5^. Therefore, we tested the influence of acute bilateral inactivation or acute functional disconnection of either amygdala territory from aDLS dopaminergic mechanisms.

### BLA-to-CeN shift in the control over cocaine-seeking habits

**Figure 2:**
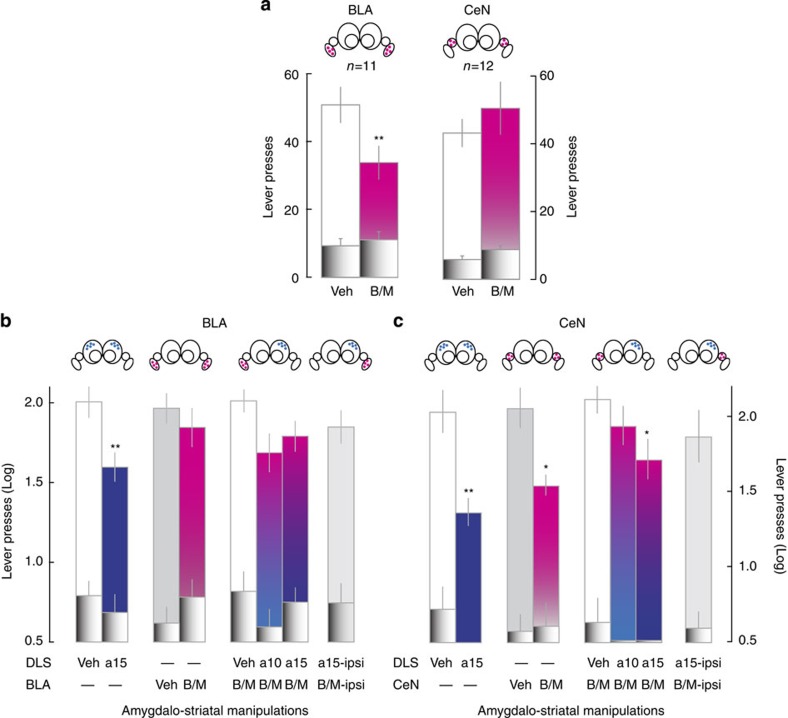
Basolateral to central amygdala shift in the control over aDLS dopamine-dependent cocaine seeking habits. (**a**) Active lever presses during early stages cocaine seeking were decreased by B/M infusions into the BLA, but not the CeN (main effect of lever: F_1,21_=138.55, *P*<0.001, partial *η*^2^=0.86; dose × structure interaction: F_1,21_=7.54, *P*<0.013, partial *η*^2^=0.26 and dose × lever × structure interaction: F_1,21_=7.01, *P*<0.015, partial *η*^2^=0.25). When cocaine-seeking behaviour was well-established, bilateral infusions of α-flupenthixol (**a**) into the aDLS decreased active lever presses both in the BLA (**b**) and CeN (**c**) groups (main effect of lever: F_1,21_=173.82, *P*<0.001, partial *η*^2^=0.89; dose: F_1,21_=34.19, *P*<0.001, partial *η*^2^=0.61; dose × lever interaction: F_1,21_=9.07, *P*<0.01, partial *η*^2^=0.30 but no dose × lever × structure interaction: F_1,21_<1). However, active lever presses were decreased both by bilateral inactivation of the CeN and functional disconnection of the CeN and aDLS (main effect of lever: F_1,10_=148.49, *P*<0.001, partial *η*^2^=0.93; manipulation: F_4,40_=3.32, *P*<0.02, partial *η*^2^=0.25 and lever × manipulation interaction: F_4,40_=3.045, *P*<0.05, partial *η*^2^=0.23) to the same extent as by bilateral aDLS α-flupenthixol infusions (Fs_1,10_<1) (**c**). In marked contrast, bilateral inactivation of the BLA or functional disconnections of the BLA and the aDLS had no influence on active lever presses when cocaine seeking was established as a habit (main effect of lever: F_1,11_=357.80, *P*<0.001, partial *η*^2^=0.97; manipulation: F_4,44_=1.17, *P*>0.16, partial *η*^2^=0.13 and lever × manipulation interaction: F_4,44_=1.67, *P*>17, partial *η*^2^=0.13). *: different from vehicle, *P*<0.05; **: different from vehicle, *P*<0.01, Newman–Keuls *post hoc* test. Pink fills represent intra-amygdala B/M infusion. Blue fills represent intra-aDLS infusions of α-flupenthixol. B/M, baclofen/muscimol mixture. Inactive lever presses are represented by black shaded fills.

Transient inactivation of the BLA by bilateral infusion of a mixture of GABA receptor agonists (baclofen/muscimol (B/M))^50^ decreased early, but not well-established cocaine-seeking behaviour (Fig. 2a), the latter being dependent on aDLS dopaminergic processes (Fig. 2b). These drug-seeking habits were not influenced by an acute disconnection of the BLA and aDLS dopaminergic mechanisms, nor were they decreased by a double ipsilateral manipulation of the two structures (Fig. 2b). In marked contrast, bilateral inactivation of the CeN, which had no effect on early cocaine seeking (Fig. 2a), dramatically decreased well-established cocaine seeking to the same extent as bilateral blockade of aDLS-dopamine receptors (Fig. 2c). This habitual cocaine seeking was also dose-dependently decreased by a functional disconnection of the CeN and aDLS, while a double ipsilateral manipulation of these two structures had no effect on instrumental responding (Fig. 2c).

Taken together these results strongly support the hypothesis that the BLA is required to recruit aDLS-dependent cue-controlled cocaine-seeking habits^5^, whereas the CeN is necessary to maintain them.

### BLA functionally regulates neural activity in the DLS

While functional interactions between the CeN and the DLS have previously been shown to support habitual control over behaviour^48^, demonstration of the functional recruitment by the BLA of aDLS-dopamine-dependent mechanisms is unprecedented. We therefore sought further to investigate the nature of the control exerted by the BLA over aDLS function.

We hypothesized that, for the BLA to trigger the instantiation of dopamine-dependent aDLS control over cocaine seeking in drug naïve animals (in which the amygdalo-striatal system is in the same state as that in experiment 1 when rats received pretraining lesions of the BLA), the BLA should influence the electrophysiological activity of aDLS neurons and this in turn should depend on antecedent glutamatergic mechanisms in the AcbC, the primary striatal target of the network originating from the BLA^8^,^11^,^29^.

**Figure 3:**
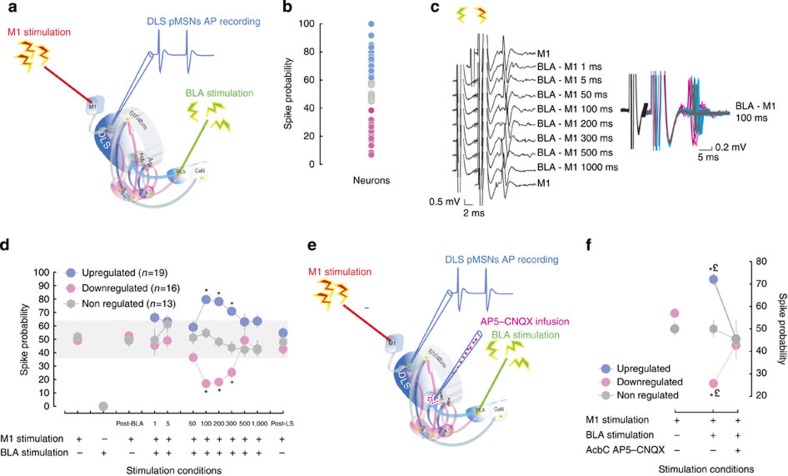
Basolateral amygdala gates the activity of pMSNs in the aDLS via glutamatergic mechanisms in nucleus accumbens core. (**a**) Schematic representation of the preparation used for the extracellular electrophysiological recordings (adapted from ref. 5). Stimulating electrodes were placed in the motor cortex (M1) and the basolateral amygdala (BLA) while a recording electrode was inserted in the aDLS where action potentials of putative medium spiny neurons (pMSNs) were recorded. The influence of BLA stimulation before M1 stimulation over the spike probability of aDLS pMSNs was measured at various interstimulation intervals (IsI). A cluster analysis (**b**) performed on the spike probability following the 100–300 ms IsI of ∼50 aDLS pMSNs was recorded, in which all displayed similar M1-stimulation-evoked action potentials, with similar spike latencies and duration (**c** and see Supplementary Fig. 7), revealed that for IsI ranging from 100 to 300 ms one subset of neurons showed a facilitation of firing (upregulated neurons, *n*=19), a second subset (downregulated neurons, *n*=16) showed a robust inhibition while a last subpopulation showed no response to BLA stimulation (non-regulated, *n*=13) (main effect of neuron class: F_2,45_=50.58, *P*<0.001, partial *η*^2^=0.69 and IsI × neuronal class interaction: F_18,405_=12.43, *P*<0.001, partial *η*^2^=0.35) (**d**). BLA stimulation alone triggered no AP in aDLS pMSNs (**d**) nor did BLA stimulation 1, 5 or 50 ms before M1 stimulation (main effect of stimulation F_3,111_=1.58, *P*>0.19, partial *η*^2^=0.04 and stimulation × neuron class interaction: F_6,111_=2.06, *P*>0.05, partial *η*^2^=0.1) However, when the BLA was stimulated 100, 200 or 300 ms before M1 stimulation, it induced a marked (that is, beyond the s.d. of the distribution of spike probability of pMSNs measured at the 1 ms IsI, displayed in grey) increase, or decrease, in the spike probability of upregulated or downregulated neurons, respectively (all *P*s<0.05 versus M1 stimulation, Dunnet *post hoc* test) (**d**). This differential effect was not observed for longer latencies (500 or 1,000 ms) (*P*s>0.05) and did not result in recurrent network effects as post-challenge M1 stimulation alone resulted in aDLS pMSNs spike probabilities that were similar to baseline, pre BLA stimulation, levels (all *P*s>0.05) (**d**). The remote control exerted by the BLA over aDLS pMSNs activity was dependent on antecedent glutamatergic transmission because it was abolished by a concomitant infusion of glutamate receptor antagonists into the AcbC (**e**) (main effect of neuron class × treatment interaction: F_4,18_=9.38, *P*<0.001, partial *η*^2^=0.67) (**f**). Thus while the BLA-modulated firing probability of up-, down-, and non-regulated aDLS pMSNs (72%, 25.7% and 50%, respectively, averaged across three IsI measurements/neuron) differed from each other before AcbC glutamate receptor blockade, they all fell to a spike probability of an ∼50% after the intra-AcbC infusion of a AP5–CNQX mixture (averaged across three IsI measurements per neuron), thereby not differing anymore from M1 stimulation-alone condition (all *P*s<0.05, Newman–Keuls *post hoc* test) (**f**). *P<0.05 versus baseline (M1 stimulation-alone condition). £: different from up- or downregulated neurons. AcbC, nucleus accumbens core; AcbS, nucleus accumbens shell; BLA, basolateral amygdala; CeN, central nucleus of the amygdala; post-BLA, M1 stimulation following BLA stimulation alone; post-LS, post Latin-square, M1 stimulation following the BLA–M1 co-stimulation protocol performed according to a Latin-square design.

For this we used extracellular electrophysiological recordings of putative medium spiny neurons (pMSNs) in the aDLS of anaesthetized rats and measured the influence of electrical stimulation of the ipsilateral BLA^51^ at various intervals (from 1 to 1,000 ms, see Fig. 3a) on the response probability of aDLS pMSNs to the stimulation of their primary glutamatergic input from the motor cortex 1 (M1)^52^,^53^ (Fig. 3a). As there are no direct projections between the BLA and the DLS^54^, we predicted that if the BLA indeed exerts control over aDLS pMSNs activity independently of the CeN, which is not involved in the early stages of intrastriatal shifts, then its gating of pMSN responses would require a multisynaptic route^5^ and would be dependent on antecedent glutamatergic mechanisms in the AcbC, its primary striatal target^11^ which has no direct connections with the CeN^55^.

Of the ∼50 aDLS pMSNs recorded, spike probability was significantly influenced by BLA stimulation (Fig. 3a, see Methods) at long, but not short, intervals prior to M1 stimulation (Fig. 3a–d). A cluster analysis (Fig. 3b, c, see Methods for further details) revealed that one subset of neurons showed a facilitation of cortico-striatal responses (upregulated, 39.6% of the population), a second subset (33.3% of the population) showed a robust inhibition while a third subpopulation showed no modulation of their cortico-striatal responses following BLA stimulation (27%) (Fig. 3d). These neurons displayed similar M1-stimulation-evoked action potentials, with similar spike latencies and duration parameters that were not influenced by BLA stimulation (all Fs<1) (Fig. 3c, Supplementary Fig. 6A,B and 7). As predicted by a lack of direct projection from the BLA to the aDLS, BLA stimulation alone triggered no AP in aDLS pMSNs (Fig. 3d) nor did BLA stimulation 1, 5 or 50 ms prior to M1 stimulation. This demonstrates that the BLA exerts no influence over aDLS pMSN activity through neural pathways that require few synapses (see ref. 51), such as the one involving the CeN.

However, when the BLA was stimulated 100, 200 or 300 ms prior to M1 stimulation, it induced a marked increase, or decrease, in the spike probability of upregulated or downregulated neurons, respectively (Fig. 3d). This differential effect was not observed for longer latencies and did not result in recurrent network effects as post-challenge M1 stimulation alone resulted in aDLS pMSN spike probabilities that were similar to pre BLA stimulation baseline levels (Fig. 3d).

### BLA controls aDLS pMSNs via AcbC glutamatergic mechanisms

These observations demonstrate that the BLA is functionally associated with the aDLS despite the absence of direct neuronal connectivity reported previously^28^. It is thereby able to exert control over the physiological activity of pMSNs in the aDLS through a multisynaptic route by modulating the spike probability of these neurons. The long BLA–M1 interstimulation interval (IsI) at which the effect is observed, alongside the differential nature of the BLA control over aDLS pMSNs, that is, an up- or downregulation of the firing probability of different populations, suggest that this effect may depend on the ascending striato-nigro-striatal dopamine-dependent loops that allow the activity of the AcbC to regulate dopamine transmission in the aDLS^14^. This hypothesis was further supported by the demonstration that the BLA control over aDLS pMSNs was abolished by glutamate receptor blockade in the AbcC (Fig. 3e-f). Indeed, an infusion of an AP5 (2-amino-5-phosphonopentanoic acid)–CNQX (6-cyano-7-nitroquinoxaline-2,3-dione) mixture into the AcbC prior to BLA–M1 stimulation in the range of 100–300 ms IsI (Fig. 3e) completely abolished the increase and decrease in spike probability observed in up- and downregulated neurons, respectively (Fig. 3f). Thus all neurons then displayed a spike probability of ∼50%, being no longer different from the one set prior to any BLA–M1 co-stimulation. This result thereby demonstrates that the control exerted by the BLA over the function of aDLS pMSNS is dependent on antecedent glumatergic transmission in the AcbC.

## Discussion

Together these results demonstrate that associative processing in two domains of the amygdala, namely the BLA and the CeN, are both necessary remotely to engage, through parallel processes, functional shifts in the striatal locus supporting well-established cocaine-seeking behaviour.

The influence exerted by the BLA over aDLS pMSNs is bidirectional, potentially reflecting a dopamine-dependent modulation of D1 and D2 dopamine receptor containing neurons. Because this influence requires antecedent glutamatergic transmission in the AcbC, it may reflect the recruitment of the striato-nigro-striatal serial, or loop, circuitry^13^,^25^, providing an informational route whereby conditioned incentive processing in the BLA reaches, and controls, aDLS-dopamine-dependent instrumental response mechanisms. This observation warrants further research to understand the routes by which the BLA and CeN control the activity of aDLS MSNs in the course of the development of cocaine-seeking habits in behaving rats.

These results also demonstrate that a shift occurs within the amygdala from the BLA to the CeN to support well-established cocaine seeking. Thus, while the BLA is necessary to recruit aDLS-dopamine-dependent mechanisms, the CeN comes online later to maintain aDLS-dependent drug-seeking habits at a point when BLA manipulations are without effect. Thus, this parallel processing in the amygdala^56^ may provide two different informational routes whereby conditioned incentive processes control the aDLS-dopamine-dependent habit system^5^.

Future studies will be necessary to define the precise circuitry supporting these amygdalo-striatal interactions^57^, as well as the mechanisms influencing the shift from BLA-to-CeN control over aDLS mechanisms in the development of cocaine-seeking habits, which may depend on top-down influences of structures such as the infralimbic or insula cortex^58^,^59^.

However, the present studies provide clear evidence for independent amydgalo-striatal functional networks linking associative processes in the amygdala, that underlie the motivational salience of drug CSs, to the rigid habit system, resulting in the emergence and persistent performance of maladaptive cocaine-seeking habits^5^.

## Methods

### Behavioural studies

Male Lister Hooded rats (Charles River Laboratories, Kent, UK) weighing 396±5 g were singly housed under a 12-h reverse light/dark cycle (off 0700 hours). Following recovery from surgery and throughout the experiment, rats were fed 20 g chow per day with water available *ad libitum*. Experiments were performed 5–7 days per week during the dark phase. All procedures were conducted in accordance with the United Kingdom 1986 Animals (Scientific Procedures) Act, Project License 80/2234. The experiments were carried out with approval from the Home Office.

### Drugs

Cocaine hydrochloride (Macfarlan-Smith, Edinburgh, UK) and α-flupenthixol (Sigma-Aldrich, Poole, UK) were prepared as previously described^13^. Quinolinic acid (Sigma-Aldrich) was dissolved in sterile phosphate-buffered saline (PBS) and infused at 0.09 M, pH=7.4. Ibotenic acid (Abcam Biochemicals, Cambridge, UK) was dissolved in PBS at a concentration of 10 μg μl^−1^, pH=7.4 . The mixture of the GABA-B and GABA-A receptor agonists, baclofen, muscimol (B/M) (Sigma-Aldrich) was dissolved in PBS at the final concentration of 0.6 and 0.06 mM^50^. The selective D1 and D2 receptor antagonists SCH23390 and raclopride (Sigma-Aldrich) were dissolved in PBS at the concentration of 0.5, 1 or 1.5 μg μl^−1^ and 1, 2 and 3 μg μl^−1^, respectively. Drug doses are reported in the salt form.

### Apparatus

Experiments were conducted using 12 standard operant conditioning chambers (Med Associates, St Albans, VT, USA) each fitted with a syringe pump and equipped with 2 cue lights above 2 levers and a houselight on the opposite wall, and were housed in sound- and light-attenuating cubicles as described previously^41^. Personal computers with Whisker software (Cardinal and Aitken, http://www.whiskercontrol.com) controlled infusions and light presentations and recorded lever presses.

### Functional disconnections

The three behavioural experiments illustrated in Supplementary Fig. 1 aimed to test the effect of functionally disconnecting either the BLA or the CeN from dopaminergic transmission in the aDLS. The rationale for using such causal manipulations is provided in Supplementary Fig. 1.

### Surgery

All rats were anaesthetized with a mixture of ketamine hydrochloride (100 mg kg^−1^; Ketaset; Fort Dodge Animal Health Ltd, Southampton, UK) and xylazine (12 mg kg^−1^; Rompun; Bayer, Wuppertal, Germany) and implanted with an intravenous jugular catheter (Camcaths, Ely, UK) as described previously^41^,^60^. Rats were then immediately positioned in a stereotaxic frame (David Kopf Instruments, Tujunga, CA, USA) with the incisor bar set at −3.3 mm (ref. 61).

For experiment 1, rats were given either a unilateral BLA lesion, a unilateral CeN lesion or a sham lesion (couterbalanced). BLA lesions were made using two infusions (anteroposterior (AP)−2.3/−3, mediolateral (ML)+/−4.6, dorsoventral (DV)−7.3 (ref. 62)) of quinolinic acid (0.3 μl per 2 min followed by a 2-min diffusion time). CeN lesions were made using one infusion (AP−2.3, ML+/−4.2, DV−7.7 (ref. 63)) of ibotenic acid (0.25 μl per 6 min^−1^ followed by a 2-min diffusion time). AP and ML coordinates were measured from bregma, DV for BLA and CeN lesions were measured from dura and skull, respectively. All rats were subsequently implanted bilaterally with 22-gauge guide cannulae (Plastics One, Roanoke, VA, USA) positioned to lie 2 mm above the aDLS (AP+1.2, ML±3, DV−3)^41^ infusion target.

For experiment 2, rats were implanted with four 22-gauge guide cannulae, all bilaterally targeting the aDLS (AP+1.2, ML±3, DV−3) and then either bilaterally targeting the CeN (AP−2.3, ML±4, DV−3.7) or the BLA (AP−2.6, ML±4.6, DV−3.6). AP and ML coordinates were measured from bregma, DV from skull.

For Experiment 3, rats were then implanted bilaterally with 22-gauge guide cannulae positioned to lie 2 mm above the aDLS (AP+1.2, ML±3, DV−3 (ref. 40)).

Cannulae and catheters were secured and maintained as described previously^40^,^41^. Rats were treated daily from the day before to 7 days after surgery with 10 mg kg^−1^ s.c. of the antibiotic, Baytril (Bayer).

### Intrastriatal and intra-amygdala infusions

Intrastriatal infusions (0.5 μl per side; bilateral for control group; unilateral for BLA-lesioned and CeN-lesioned groups—see Supplementary Fig. 2) were made via 28-gauge steel hypodermic injectors (Plastics One) lowered to the injection sites 2 mm (aDLS), 4 mm (CeN) or 5 mm (BLA) ventral to the end of the guide cannulae.

α-flupenthixol, SCH23390 and raclopride were, respectively, administered at the doses of 10 or 15 μg per infusion; 0.25, 0.5 and.75 μg per side and at 0.5, 1 and 1.5 mg per side. B/M was administered at 0.3/0.03 nmol per infusion, respectively. Infusions were made over 90 s using a syringe pump (Harvard Apparatus, Holliston, MA, USA) and were followed by a 60-s period to allow diffusion of the infused drug or vehicle before injectors were removed and obturators were replaced. Test sessions (see Procedures) began 5 min later.

For all pharmacological challenges, intracranial infusions were delivered according to a Latin-square design and were separated by at least two baseline sessions to control that stable levels of cocaine seeking or cocaine self-administration were maintained throughout.

### Procedures

For all the experimental groups, cocaine self-administration training sessions began 7 days following surgery (Supplementary Fig. 1). Cocaine (0.25 mg per infusion; 0.1 ml per 5 s^−1^) was available under a continuous reinforcement (that is, fixed-ratio 1 (FR1)) schedule. One active lever press resulted in an infusion and initiated a 20-s time out. Each cocaine infusion was accompanied by a 20-s illumination of the active lever-specific cue-light (CS). The houselight was extinguished and both levers were retracted during the time out. Pressing on the inactive lever was recorded but had no programmed consequence. The maximum number of available cocaine infusions during this stage was 30. Active and inactive lever assignment was counterbalanced. All test conditions were administered in a counterbalanced, Latin-square order of treatment, and sessions were conducted prior to, and were thus unaffected by, self-administered cocaine on that day (Fig. 1). Each test was immediate followed by the appropriate training session.

For Experiment 1 (Supplementary Fig. 1 top panel), following 10 training sessions under FR1, the effects of aDLS-dopamine receptor blockade on early-stage cocaine seeking were tested. Infusions (bilateral for control group, unilateral and contralateral to the lesion in BLA and CeN groups) of α-flupenthixol (0 and 10 μg per infusion) were made into the aDLS. During each 15-min test session, every active lever press resulted in a 1-s light CS presentation, and cocaine was only delivered on the first lever press following the 15-min interval (that is, FI15 (FR1:S)). Thus, the early-performance tests were conducted prior to, and were thus unaffected by, self-administered cocaine on that day (Fig. 1). Each test session was immediately followed by a FR1 cocaine self-administration training session (that is, up to 30 reinforcers in a 2-h session).

Following the early-performance cocaine-seeking tests, the response requirement was increased across the daily training sessions from FR1 to FR3, FR5(FR2:S), FR10(FR2:S) and ultimately FR10(FR4:S)^41^. Under each intermediate second-order schedule, completion of the unit schedule (given within parentheses) resulted in a 1-s CS light presentation; cocaine infusion and the 20-s time out were given only on completion of the overall schedule. Therefore, for the intermediate stage assessments, rats had been trained under conditions in which contingent presentations of the cocaine-associated CS occurred after 4 responses (FR4:S); cocaine was delivered on completion of the tenth set of 4 lever presses, that is, 40 lever presses. After reaching the FR10(FR4:S) schedule, rats began the transition-stage cocaine-seeking tests. During each 15-min test session with aDLS α-flupenthixol infusions (0 and 10 μg per infusion), every four active lever presses resulted in a 1-s light CS presentation, and cocaine was only delivered on the fourth lever press following the 15-min interval (that is, FI15(FR4:S)). Thus, the transition-stage performance tests were again conducted prior to, and were unaffected by, daily self-administered cocaine. Each test session was immediately followed an FR10(FR4:S) cocaine self-administration training session (30 reinforcers over 2 h).

The response requirements were then again increased through daily training sessions across the following reinforcement SOCR:FR10(FR6:S), FR10(FR10:S) and finally to an overall schedule of FI15(FR10:S)^13^,^40^,^41^ (Supplementary Fig. 1) during which responding was maintained by contingent presentation of the cocaine-associated Cs every tenth lever press (FR10:S), and each of the five daily cocaine infusions was delivered after the tenth lever press following completion of each 15-min fixed interval. Following 15 sessions, the habitual-stage tests were conducted in which the effects of aDLS α-flupenthixol infusions (0, 5, 10 and 15 μg per infusion) were assessed. The first 15-min interval of the schedule provides a time period in which no cocaine has been administered, yet rats are actively seeking cocaine.

Finally, on completion of contralateral testing rats were assessed for effects of ipsilateral aDLS-dopamine receptor blockade (0 and 10 μg per infusion) (Supplementary Fig. 5). Rats in the BLA and CeN groups were given infusions in the aDLS on the same side of the brain as their unilateral amygdala lesion; rats in the control group were given unilateral aDLS infusions.

For Experiment 2 (Supplementary Fig. 1 middle panel), following five training sessions under FR1 the effects of amygdala inactivation on early-stage cocaine seeking were tested. Bilateral infusions into the amygdala (either the CeN or the BLA depending on the group) of B/M^22^ or PBS (counterbalanced) were made. Dopamine receptor blockade in the aDLS was not assessed at this stage as it has already been established that it has no effect on cocaine-seeking at this point^40^,^41^.

The response requirement was then increased across the daily training sessions as described for Experiment 1 all the way to FI15(FR10:S). Following 15 sessions of training under this schedule, habitual-stage tests were conducted. Rats received bilateral inactivation (via B/M) of the amygdala (CeN or BLA), bilateral dopamine receptor blockade (α-flupenthixol, 10 μg per side) in the aDLS, a disconnection procedure involving unilateral amygdala inactivation with contralateral aDLS-dopamine receptor blockade and an ipsilateral procedure in which both the amygdala and aDLS were treated in the same hemisphere. For each of these tests, PBS control infusions were intermixed.

For Experiment 3 (Supplementary Fig. 1 bottom panel), following 10 sessions under FR1 rats were trained under fixed interval SOCR with daily increases in the interval duration, from 1 to 15 min as previously described^13^. Rats were then trained for 20 sessions under the FI15(FR10:S) schedule prior to being challenged with bilateral intra-aDLS infusions of vehicle, SCH23390 or raclopride.

### Histology

Histology was conducted as described previously^40^,^41^.

### Electrophysiological study

Despite the different time course in the functional control over the recruitment of aDLS-dependent cue-controlled cocaine cocaine-seeking behaviour between the BLA and the CeN and their dissociable neural targets, we wanted to identify better the neural pathway whereby the BLA controls aDLS function. Such remote functional connectivity having never been tested before, we decided to use extracellular electrophysiological recordings of aDLS medium spiny neurons to measure the nature and the characteristics of the influence of BLA stimulation over the spike probability of these neurons.

### Animals

Male Sprague–Dawley rats (Charles River, France) weighing 300 g at experiment start were housed two per cage under conditions similar to the behavioural experiments.

### Surgery

Rats were anesthetized by a single i.p. injection of urethane (1.7 g kg^−1^, in distilled water). Rats were positioned in a stereotaxic frame (m2e-Unimecanique, France) and maintained at 37 °C with a homeothermic blanket. Burr holes were drilled in the cleaned skull above the electrode targets, and the overlying dura was carefully resected. The coordinates are relative to bregma in the right hemisphere.

### Stimulating electrodes

A concentric electrode (SNEX-100, Phymep) was implanted in M1 (AP+4.15; ML+3 ; DV−2.5). A current pulse (0.5-ms long) was applied by an isolated stimulator (DS3 digitimer) triggered by a 1401 Plus system (Cambridge Electronic Design, Cambridge, UK). Because the target aDLS MSNs are silent, cortical stimulation was continuously applied at 0.3 Hz. A bipolar electrode (SNEX-200, Phymep) was lowered into the BLA (AP−2.5; ML+5; DV−7.3). The two poles of the electrodes are oriented in the same sagittal plan.

### Electrophysiological recordings and neuron selection

The recording electrode was lowered to the aDLS (AP+1.8; ML+3, from DV−3.3 to DV−4.4) ipsilateral to the stimulating electrodes. The recording electrodes were pulled from borosilicate glass capillaries (GC150F, Harvard Apparatus, UK) using a P97 micropipette puller (Sutter instrument, CA, USA). The tip was broken back under microscopic control to achieve an impedance of 10–15 MΩ as measured *in situ* with an axoclamp2B (Axon instruments, Foster City, CA) by bridge balance. The electrodes were filled with 0.4 M NaCl. Through the electrode, the extracellular potential was recorded with an axoclamp2B amplifier in the bridge mode versus a reference electrode maintained in contact with the skull skin by a sponge moistened with 0.9% NaCl. The signal was amplified 10 × by the axoclamp2B, further amplified 100 × and band pass filtered (low-pass filter at 300 Hz and high-pass filter at 20 kHz) via a differential AC amplifier (model 1700; AM systems, Carlsborg, WA). Spike occurrence was continuously recorded by a 1401 Plus Cambridge Electronic Design system running Spike 2.

The neurons of interest were isolated by slowly lowering the recording electrode in the aDLS while a single electrical stimulation pulse was delivered to M1 at an overall rate of 0.3 Hz. A total of ∼50 striatal neurons that were excited by stimulation of M1 were recorded. Because MSNs compose ∼95% of striatal neurons^64^,^65^ and because putative interneurons were not tested, almost all of the neurons recorded in this study were likely to be the medium spiny subtype identified after stimulation of the primary glutamatergic input to the DLS, namely M1. Neurons were considered as pMSNs based on their electrophysiological properties: a spike duration above 1 ms over 20 trials and the shape of their action potential, as previously described^51^,^66^,^67^ (Supplementary Fig. 6A).

Neurons with sustained spontaneous firing, which produced more than two spikes following a single electrical stimulation, with a spike duration <1 ms were considered to be putative interneurons, and were not analysed.

### BLA stimulation protocol

Once a pMSN was found, the effect of the pre-stimulation of the BLA on the M1-evoked spike probability was tested. M1 stimulation current intensity was adjusted to produce about 50% evoked spike probability (the current intensity was adjusted for each neuron). To obtain a stable baseline spiking probability, M1 stimulation was delivered for 20 trials at a rate of 0.3 Hz.

To test the effect of the BLA stimulation alone on the spike probability of the selected pMSNs, M1 stimulation was turned off and BLA stimulation was applied alone for at least 40 trials. Following these 20 trials, a single BLA stimulus pulse (.5 ms, .5 mA) was delivered before the M1 stimulation pulse for 20 trials with IsI ranging from 1 to 1,000 ms (IsI tested: 1, 5, 50, 100, 200, 300, 500, 1,000 ms). These IsI, which were applied following a Latin-square design, were chosen to test mono- and poly-synaptic delays.

### AcbC glutamate receptor blockade

AcbC glutamatergic receptors were blocked by an infusion of a mixture of the AMPA (α-amino-3-hydroxy-5-methylisoxazole-4-proprionic acid) and NMDA (N-methyl-d-aspartate) receptor antagonists—CNQX and AP5^68^,^69^. An injection electrode filled with the CNQX/APV solution (4 mg ml^−1^ and 8 mg ml^−1^, respectively) was lowered into the AcbC (AP+1.6; ML+1.4; DV−7) ipsilateral to the stimulating and recording electrodes. The injection electrode was pulled from borosilicate glass capillaries (GC100F, Harvard Apparatus) using a P97 micropipette puller. A scale system on the pipette allowed us to accurately measure each injected volume by visual observation of the meniscus. After the injection, the absence of meniscus movement showed that no spontaneous leakage occurred. The pulled tip of the injection pipette was broken under a microscopic control at an external diameter of ∼30–40 μm. A volume of 0.5 μl of CNQX/AP5 was injected into the AcbC^11^ with a picoinjector (UC-8 controller, Bioscience tools). BLA–M1 co-stimulations were thus applied and we measured the effect of an infusion of the CNQX/APV mixture into the AcbC on the influence exerted by BLA pre-stimulation over spike probability of aDLS pMSNs in response to M1 stimulation for the specific 100–200–300 ms IsI, those at which the recorded neurons in the aDLS had been shown to be sensitive to BLA stimulation prior to M1 stimulation.

### Data and statistical analyses

Data are presented as mean±s.e.m. except when individual points are displayed (Fig. 2b). Analyses were performed using Statsoft STATISTICA 10 software.

Assumptions about normality of distribution and homogeneity of variance were assessed using the Kolmogorov–Smirnov and Levene tests, respectively. In case of violation of at least one of these assumptions, data sets were log-transformed (Fig. 2b,c).

For behavioural studies, lever presses during each 15-min drug-seeking tests were analysed using two-way analyses of variance (ANOVAs) with lever, dose and training level as within-subject factors.

For electrophysiological studies, spike probability evoked by M1 stimulation was calculated as the ratio between the number of evoked APs and the number of stimulation cycles. Change in probability of spike discharge evoked by BLA stimulation was calculated by comparing the probability of spike discharge in M1 stimulation-only situation and the spike probability on each pre-stimulation condition.

A cluster analysis on average spike probability of pMSNs, obtained for the BLA–M1 IsI of 100, 200 and 300 ms, identified three neuronal populations according to the nature of BLA pre-stimulation influence on the spike probability of discharge evoked by the M1 stimulation.

Latency to AP and AP duration were calculated as previously described^66^. Briefly, the AP duration was measured as the time between start of depolarization and the depolarization of each AP evoked by M1 stimulation. AP waveforms were averaged using the spike 2 software and extracted as the shape of the APs of the recorded neuron.

When no AP was recorded in some downregulated neurons (on only four occasions), provided there was no effect of the stimulation on the AP duration and latency to first AP overall, the value was computed as the average of the other values (50, 100, 200 or 300 ms IsI). Variations in spike probability, latency to first AP and AP duration were analysed by ANOVA with neuronal type as between-subject factor and stimulation condition (including IsI) as within-subject factors.

Significance was set at *α*=0.05. Significant interactions were analysed further using the Dunnet *post hoc* or Newman–Keuls *post hoc* tests where appropriate. Tests used are stated in either the main text or figure legends. Effect sizes are reported using partial *η*^2^ values^70^.

## Additional information

**How to cite this article:** Murray, J. E. *et al*. Basolateral and central amygdala differentially recruit and maintain dorsolateral striatum-dependent cocaine-seeking habits. *Nat. Commun.* 6:10088 doi: 10.1038/ncomms10088 (2015).

## Supplementary Material

Supplementary Information Supplementary Figures 1-7 and Supplementary References
