# Supplementary material for: Basolateral and central amygdala differentially recruit and maintain dorsolateral striatum-dependent cocaine-seeking habits
Source: Nat Commun. 2015 Dec 14;6:10088. doi: 10.1038/ncomms10088 (PMC4682035; doi:10.1038/ncomms10088)
Supplement: Supplementary Information — Supplementary Figures 1-7 and Supplementary References [file ncomms10088-s1.pdf]

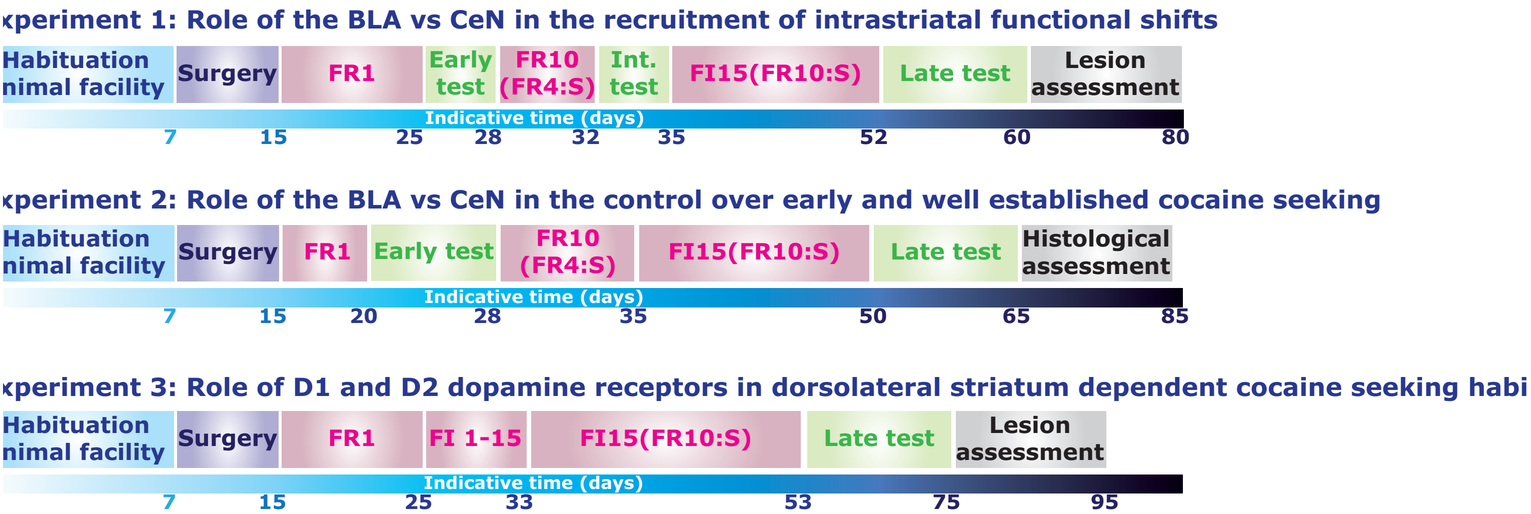

Supplementary figure 1: Timeline of self-administration experiments.

Int.: intermediate, FI: fixed-interval. See Methods for more details.

These experiments are aimed at testing the effect of a functional disconnection of either the BLA or the CeN and dopaminergic transmission in the aDLS.Functional disconnection involves asymetrical manipulations of neural structures suggested to be serially involved in a specific function. Operationally, it aims at disrupting one structure in one hemisphere and manipulating an afferent or efferent structure in the opposite hemisphere. If these structures are interrelated then disconnection results in an effect similar to bilaterally disrupting either structure alone[1]. Functional disconnections of the CeN and DLS`, which are connected via the substantia nigra pars compacta[2], have already been shown to disrupt conditioned orientation[2].
As there is no direct connectivity between the BLA and the DLS[3], serial connectivity involving intervening structures, the last of which eventually sends dopaminergic projections to the DLS, should be responsible for an effect of a functional disconnection of the BLA and dopaminergic transmission in the aDLS. A strong candidate for this connection is the AcbC activation of striato-nigro-striatal circuitry. The BLA also projects to the CeN[4], which has distinct influences on behavior compared to those of the BLA. Rats with pretraining CeN lesions do not develop normal habitual behavior[5,6], and lesions of the CeN disrupt autoshaping and general Pavlovian to instrumental transfer (PIT)[7,8], but have no impact on specific PIT[7]. The CeN has major projections to the SNc but limited projections to the VTA[9]. The CeN projections are largely to the lateral third of the posterior SNc[4,10], an area with connectivity directly to the DLS and only minimal influence on the ventral striatum[11,12]. This circuitry suggests at the very least, differential amygdalar influences on AcbC dopamine, and at the most, a potential signal bypass of the AcbC. Indeed, unlike stimulation of the BLA, stimulation of the CeN does not have measurable dopamine-evoking effects from the VTA[13], and while CeN disconnection from the SNc resulted in impaired general PIT, a disconnection from the VTA resulted in no such impairment[2], indicating that the more motivationally invigorating role for CeN is likely through the SNc-to-DLS circuitry.

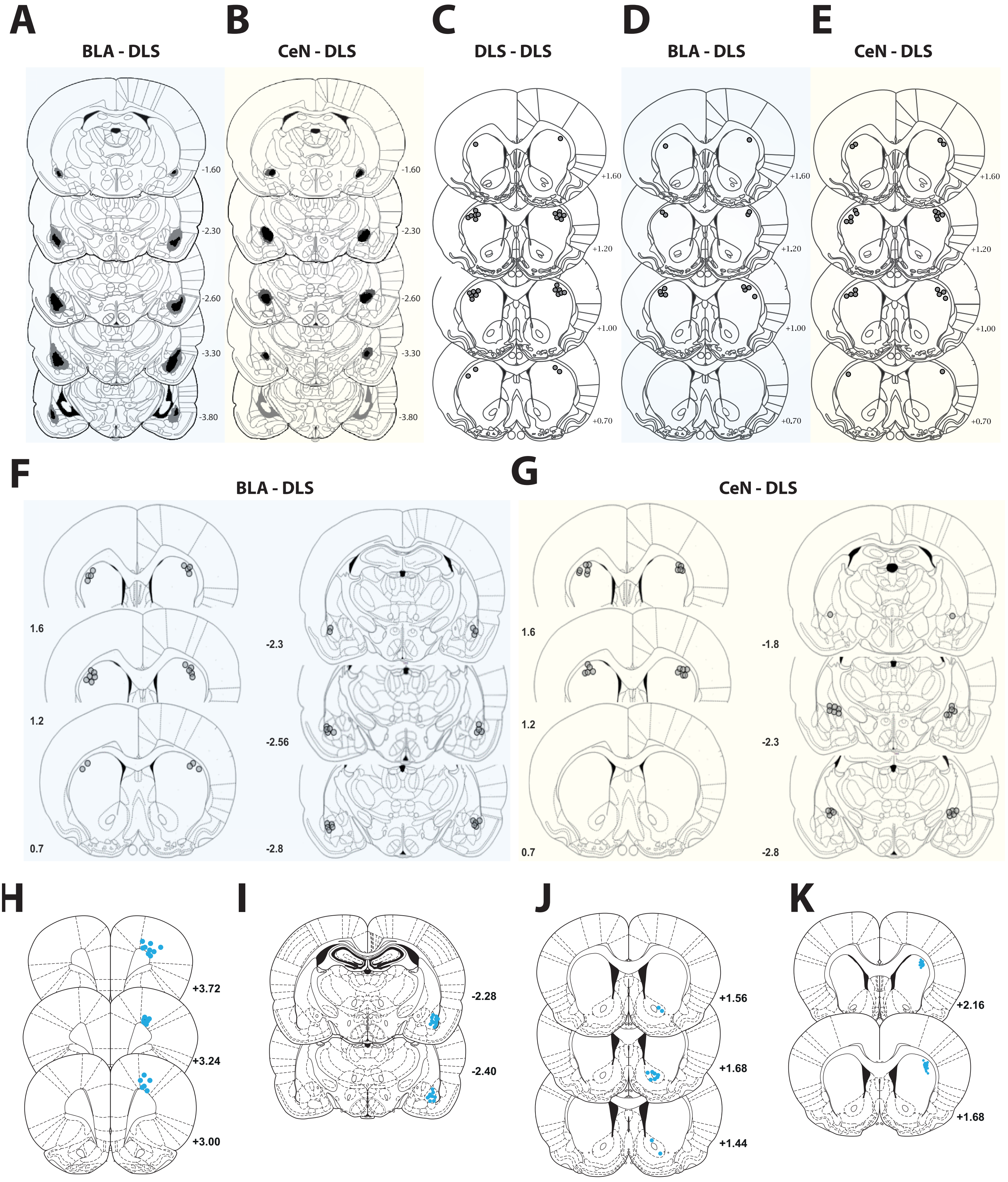

Supplementary figure 2: Histological assessment of lesions, electrodes and cannula placements.

Histological assessment was performed blind to experimental results. **Top panel.** Unilateral BLA lesioned rats (**A**) included in the behavioral analysis (n=7) displayed a lesion of the majority of the BLA that did not extend to the CeN; neuronal loss and associated gliosis extended from

approximately -1.5 to -4.5 mm relative to bregma. Likewise, unilateral CeN lesioned rats (**B**) included in the behavioral analysis (n-12) displayed a lesion of the majority of the CeN that did not extend to the BLA; neuronal loss and associated gliosis extended from approximately -1.5 to -3.5 mm relative to bregma. Schematic representations[14] of the DLS infusion locations (experiments 1 and 3) are shown in **C** (DLS-DLS Control Group), **D** (BLA-DLS Group), and **E** (CeN-DLS Group). **Middle panel.** All animals included in the behavioural statistical analyses had cannulae located bilaterally within the aDLS and BLA (**F**) or the aDLS and CeN (**G**). **Bottom panel.** For the electrophysiology experiment, stimulating electrodes were located in the motor cortex M1 (**H**) and the BLA (**I**) while injecting electrodes were all located in the AcbC (**J**) and the recording electrodes in the aDLS (**K**).

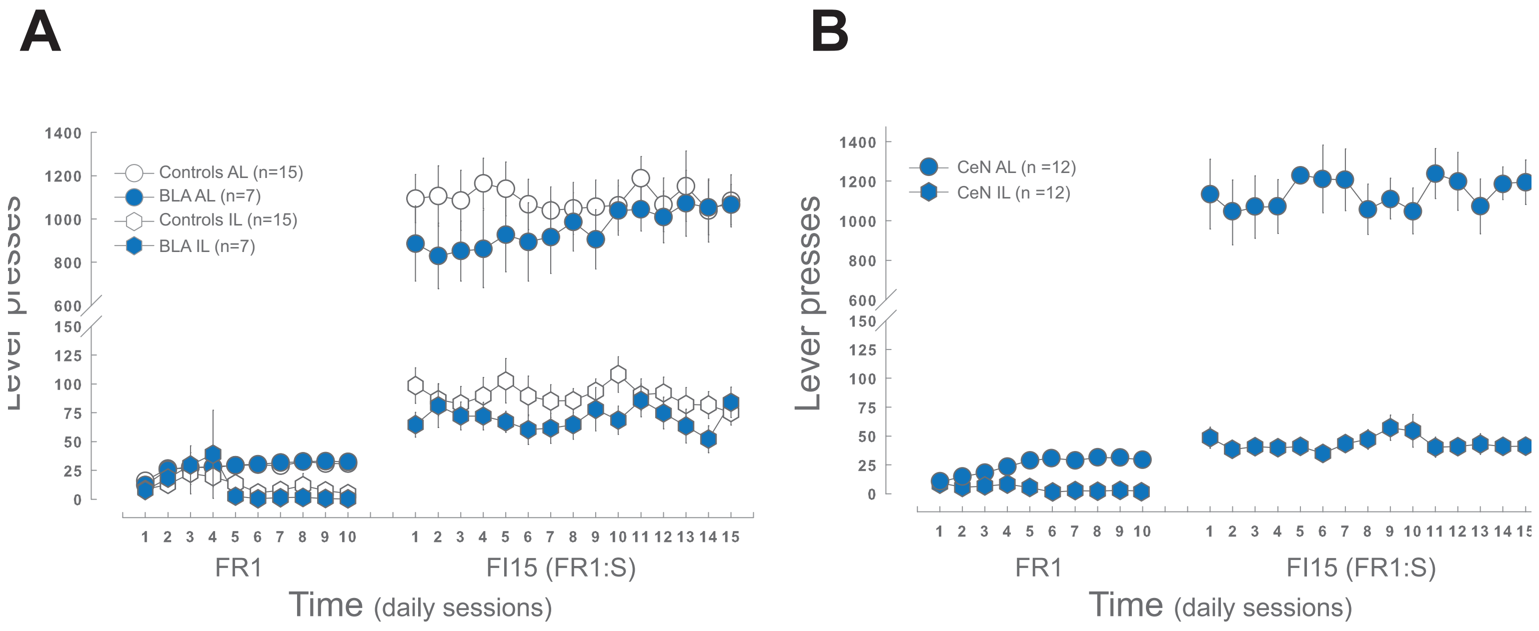

Supplementary figure 3: Unilateral lesions of the BLA or CeN did not impair the acquisition of instrumental responding for cocaine or the performance of cue-controlled cocaine seeking behaviour.

A. As compared to control rats, rats with a unilateral excitotoxic lesion of the BLA were impaired neither in the acquisition of instrumental responding for cocaine under continuous reinforcement (FR1) [main effect of lever: $F_{1,20} = 17.32$, $p < .001$; partial $\eta^2 = .46$, lever x day interaction: $F_{9,180} = 2.9156$, $p < .001$, partial $\eta^2 = .13$, but no effect of group or group x lever or group x lever x day interaction: all Fs < 1], nor in their performance of cue-controlled cocaine seeking during 15min periods under a second order schedule of reinforcement for cocaine (FI15(FR10:S)) [Main effect of lever: $F_{1,\ 20} = 108.41$, $p < .001$, partial $\eta^2 = .84$, but no effect of group, group x day, group x lever, day x lever or group x day x lever interactions all Fs < 1].

B. Similarly, a unilateral lesion of the CeN did not impair the acquisition of cocaine self-administration as revealed by a significant increase in active lever presses under FR1 [main effect of lever: $F_{1,11} = 143.34$, $p < .0001$, partial $\eta^2 = .93$, day: $F_{9,99} = 2.77$, $p < .001$, partial $\eta^2 = .2$ and day x lever interaction: $F_{9,99} = 21.33$, $p < .0001$, partial $\eta^2 = .66$] similar to the one observed in controls [main effect of group: $F_{1,25} = 3.43$, $p = .076$ and group x lever interaction: $F_{1,25} < 1$]. Similarly, a unilateral lesion of the CeN did not impair the high rates of active lever pressing under a second order schedule of reinforcement of cocaine [Main effect of lever: $F_{1,\ 11} = 91.94$, $p < .0001$, partial $\eta^2 = .89$] so that CeN lesioned rats did not differ from controls [no effect of group, group x day, group x lever, day x lever or group x day x lever interactions all Fs < 1]. AL: Active lever, IL: Inactive lever. BLA: basolateral amygdala, CeN: central nucleus of the amygdala. FR1: Fixed ratio 1., FI15: Fixed interval 15 minutes.

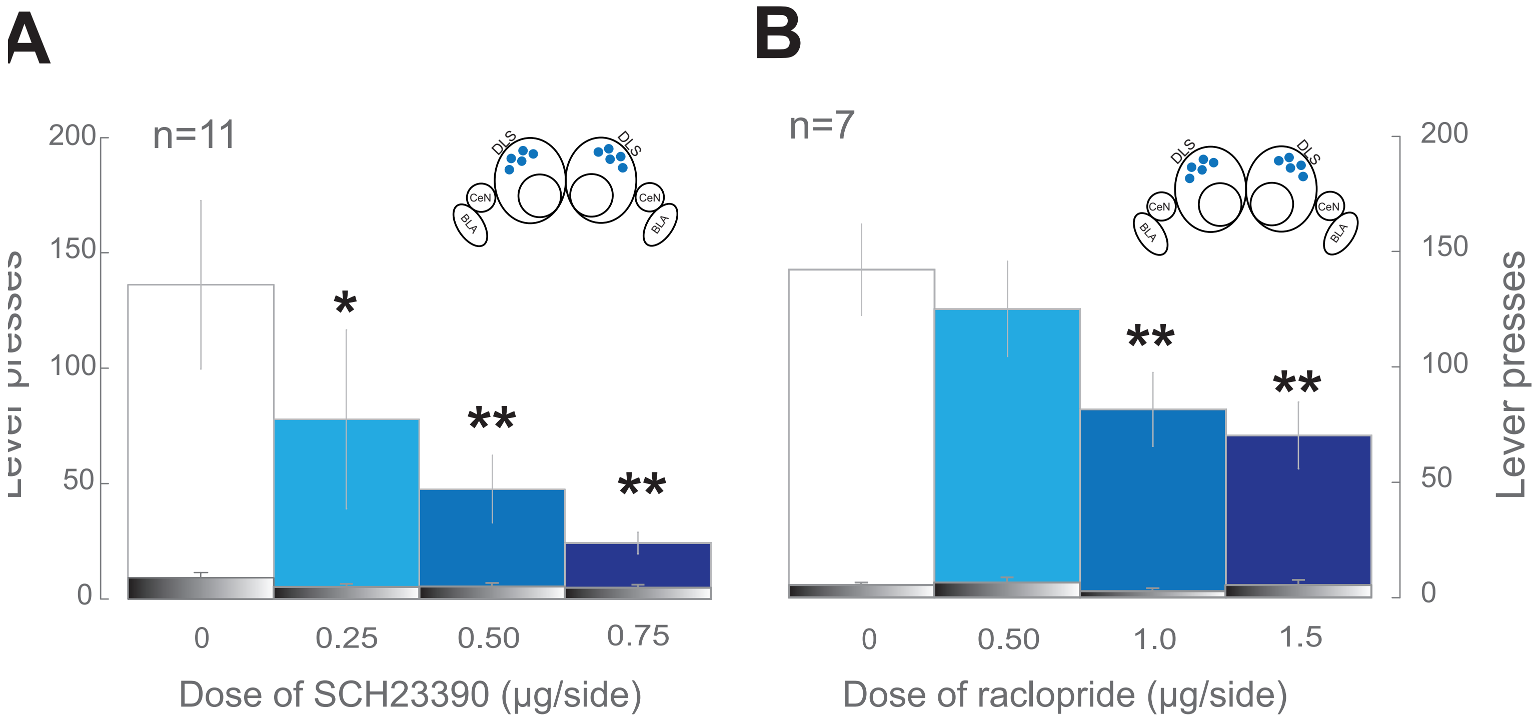

Supplementary figure 4: Involvement of dorsolateral striatum D1 and D2 dopamine receptors in the control over cocaine seeking habits

The effect of infusions of α-flupenthixol in the aDLS on well established cocaine seeking behaviour reflected effects at both D1 and D2 dopamine receptors. Thus in rats trained to seek cocaine under the control of drug-associated stimuli for more than 20 days cue-controlled cocaine seeking during the first drug-free 15 minute interval of the daily sessions was dose-depdendently reduced by intra aDLS infusions of either the selective dopamine D1 receptor antagonist SCH23390 (**A**) [main effect of dose: $F_{3,30} = 3.92$, $p < .02$ partial $\eta^2 = .26$; lever $F_{1,10} = 14.38$, $p < .005$, partial $\eta^2 = .58$, and dose x lever interaction: $F_{3,30} = 3.65$, $p < .03$, partial $\eta^2 = .26$] or the selective D2 dopamine receptor antagonist raclopride (**B**) [main effect of dose: $F_{3,18} = 7.62$, $p < .002$, partial $\eta^2 = .56$; lever: $F_{1,6} = 47.35$, $p < .001$, partial $\eta^2 = .88$ and dose x lever interaction: $F_{1,6} = 47.35$, $p < .001$, partial $\eta^2 = .56$]. Grey bars represent inactive lever presses.

*: p < .05, **: p < .01 vs vehicle, Newman Keuls post-hoc test.

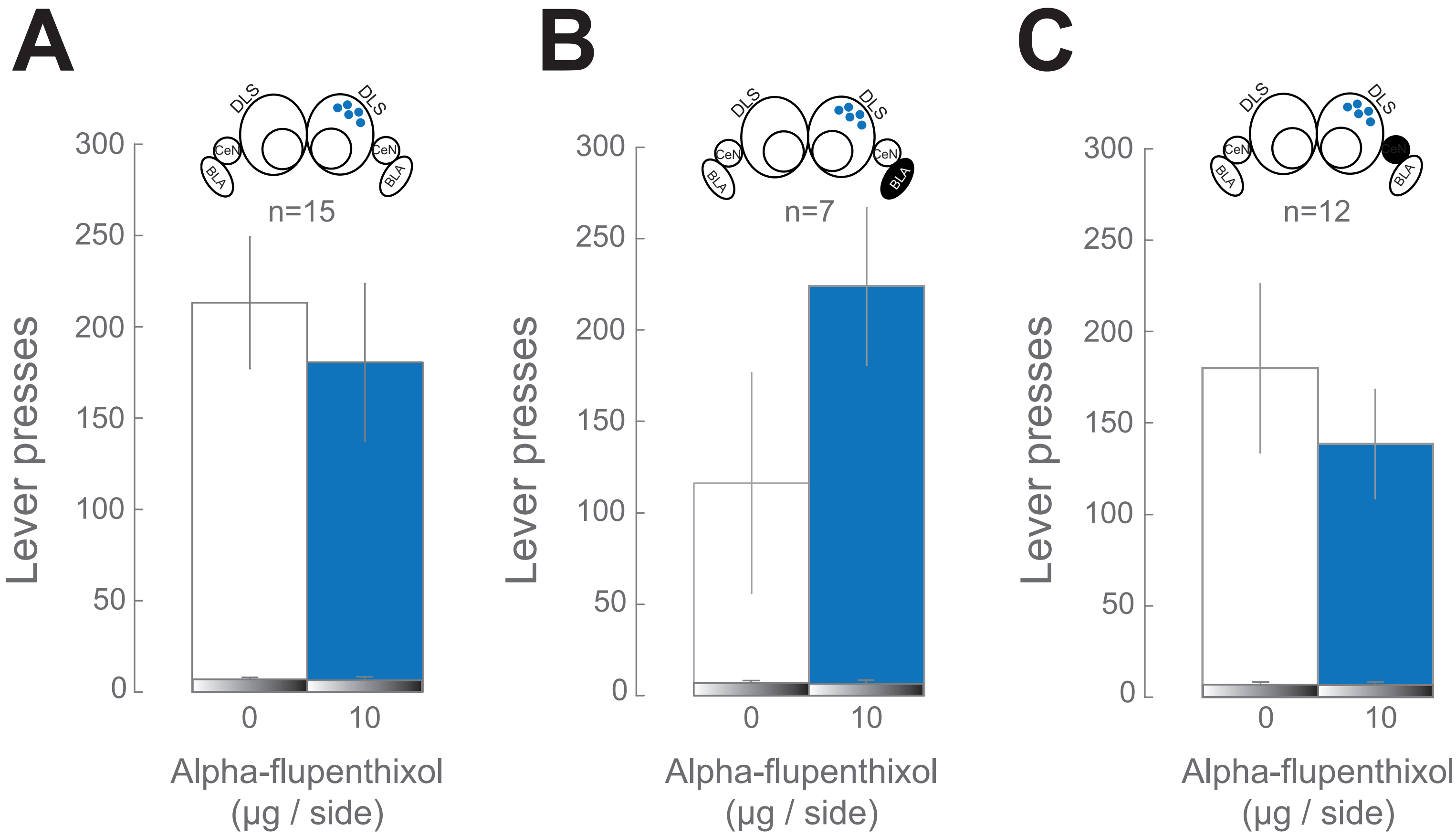

Supplementary figure 5: Double ipsilateral manipulations of amygdala-striatal networks have no effect on habitual cocaine seeking behaviour.

To further test the hypothesis that the decrease of habitual cocaine seeking behaviour observed following fuctional disconnections of either the BLA or the CeN and DLS dopamine transmission was indeed attributed to the impaired recruitment of intrastriatal functional shifts eventually devolving control over behaviour to the DLS on the lesioned side, we tested whether a unilateral α-flupenthixol infusion in the DLS-DLS Control group (**A**) or an ipsilateral α-flupenthixol infusion in the BLA-DLS (**B**) and CeN-DLS (**C**) groups would affect cocaine seeking under the same training conditions. In all groups, active lever pressing remained higher than inactive, *F*s ≥ 21.71, *p*s ≤ .001, partial $\eta^2 > .26$, but there were no effects of Dose, *F*s ≤ 1.29, *p*s ≥ .280, or Dose x Lever interactions, *F*s ≤ 1.17, *p*s ≥ .302. Inactive lever presses are represented in black.

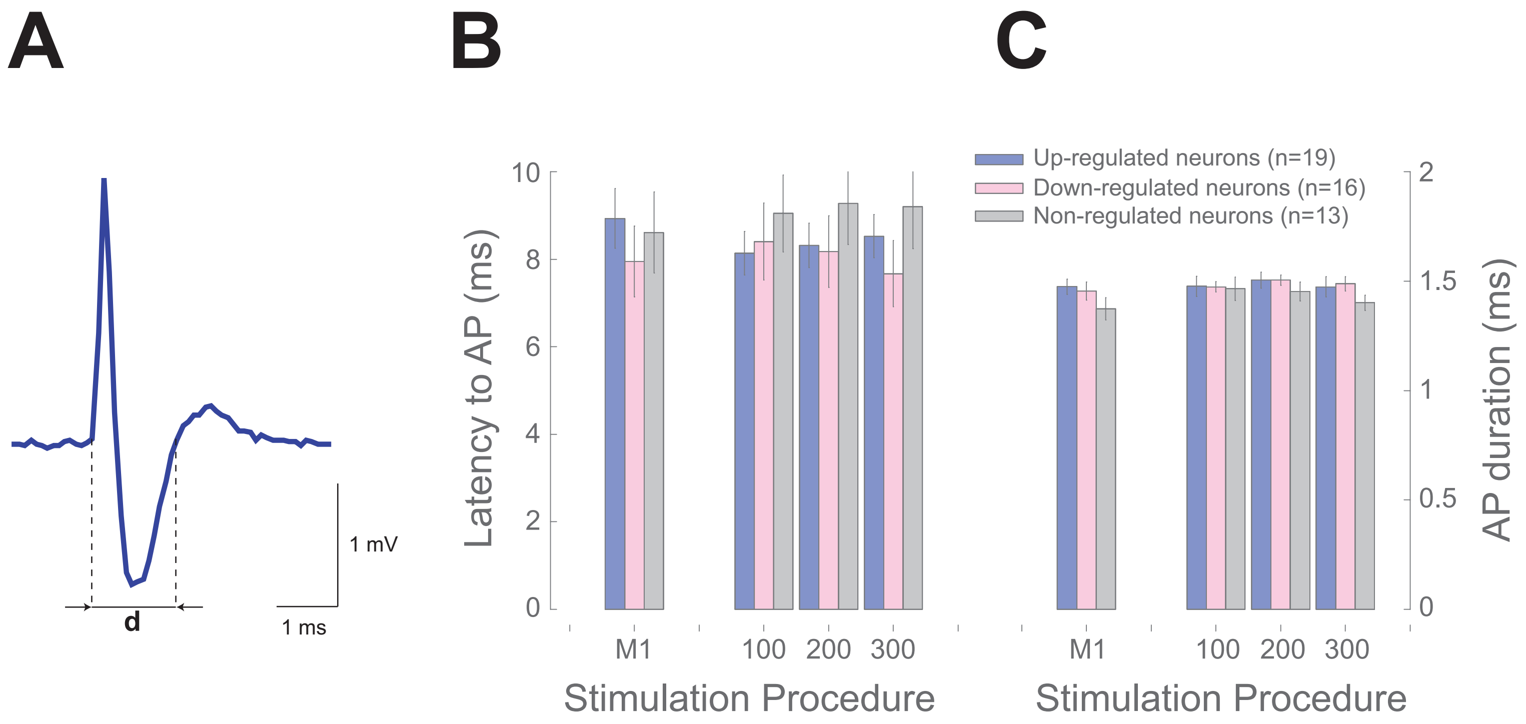

Supplementary figure 6: BLA control over DLS pMSNs spike probability influenced neither the latency to, nor the duration of, action potentials (AP).

Action potentials of recorded neurons displayed waveforms charateristic of medium spiny neurons (**A**) with an average (the trace represented here is an average of 20 events) duration (d) higher than 1 ms. The influence exerted by the BLA over the activity of DLS pMSNS was specific to spike probability and did not alter the intrinsic properties of the neurons' activity. Thus, BLA stimulation with IsI of 100, 200 or 300 ms, which resulted in increase, or decrease, in spike probability of up or down-regulated neurons, respectively, had no influence over the latency to the first action potential (AP) [main effect of stimulation: $F_{3,111} < 1$, neuronal population: $F_{2,37} <1$ and stimulation x neuronal population interaction: $F_{6,\ 111} = 1.73$, $p = .12$] (**B**) or the AP duration [main effect of stimulation: $F_{3,138} = 2.15$, $p = .096$, neuronal population: $F_{2,46} < 1$ and stimulation x neuronal population interaction: $F_{6,138} < 1$] (**C**) in either of the neuronal populations recorded in the DLS.

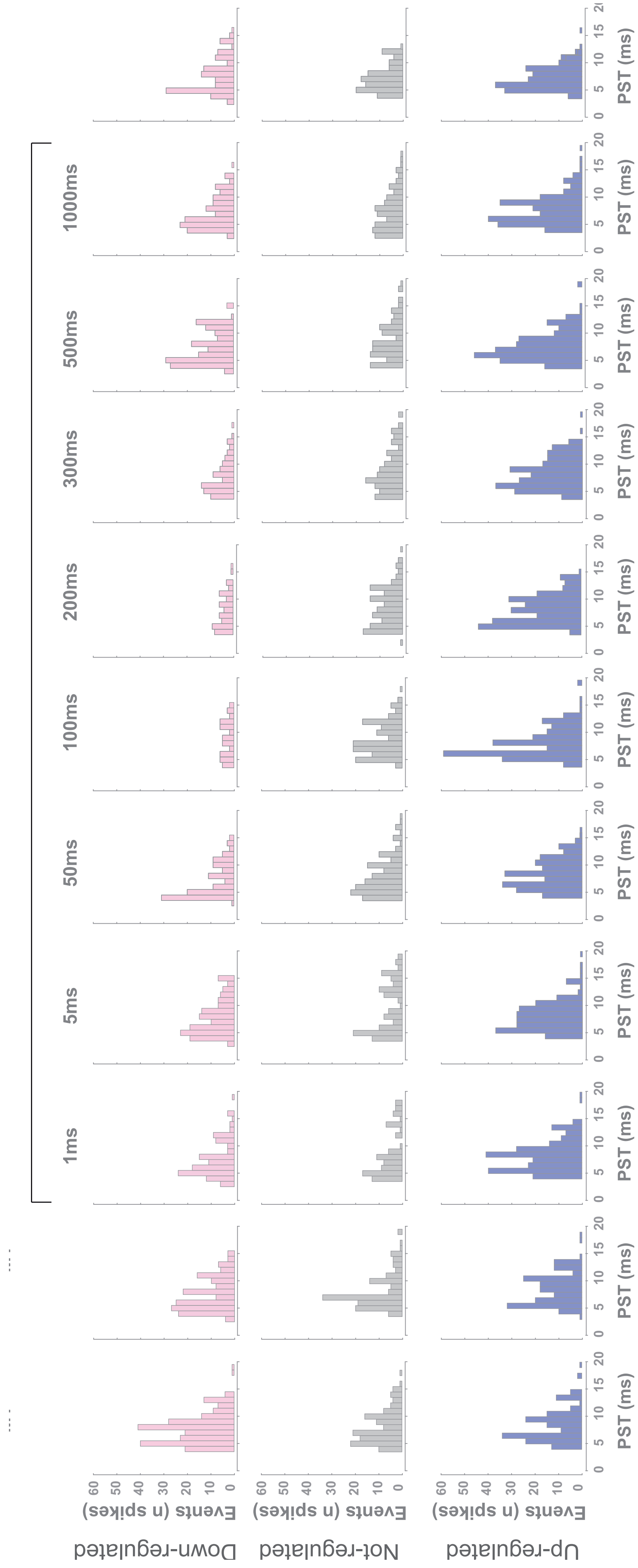

Supplementary figure 7: Peristimulation time histograms of the spike probability of down-, non-and up-regulated neurons.

Supplementary references

1 Belin, D. & Everitt, B. J. Cocaine seeking habits depend upon dopamine-dependent serial connectivity linking the ventral with the dorsal striatum. *Neuron* **57**, 432-441 (2008).

2 El-Amamy, H. & Holland, P. Dissociable effects of disconnecting amygdala central nucleus from the ventral tegmental area or substantia nigra on learned orienting and incentive motivation. *Eur J Neurosci* **25**, 1557-1567, doi:10.1111/j.1460-9568.2007.05402.x (2007).

3 Kelley, A. E., Domesick, V. B. & Nauta, W. J. The amygdalostriatal projection in the rat--an anatomical study by anterograde and retrograde tracing methods. *Neuroscience* **7**, 615-630 (1982).

4 Fudge, J. & Emiliano, A. The extended amygdala and the dopamine system: another piece of the dopamine puzzle. *J Neuropsychiatry Clin Neurosci* **15**, 306-316 (2003).

5 Hatfield, T., Han, J. S., Conley, M. & Gallagher, M. Neurotoxic Lesions of Basolateral, But Not Central, Amygdala Interfere with Pavlovian Second-Order Conditioning and Reinforcer Devaluation Effects. *Journal of Neuroscience*, 5256-5265 (1996).

6 Lingawi, N. W. & Balleine, B. W. Amygdala central nucleus interacts with dorsolateral striatum to regulate the acquisition of habits. *J Neurosci* **32**, 1073-1081, doi:10.1523/JNEUROSCI.4806-11.2012 (2012).

7 Corbit, L. H. & Balleine, B. Double dissociation of basolateral and central amygdala lesions on the general and outcome-specific forms of pavlovian-instrumental transfer. *J Neurosci* **25**, 962-970 (2005).

8 Holland, P. & Gallagher, M. Double dissociation of the effects of lesions of basolateral and central amygdala on conditioned stimulus-potentiated feeding and Pavlovian-instrumental transfer. *Eur J Neurosci* **17**, 1680-1694, doi:10.1046/j.1460-9568.2003.02585.x (2003).

9 Lee, H., Gallagher, M. & Holland, P. The central amygdala projection to the substantia nigra reflects prediction error information in appetitive conditioning. *Learn Mem* **17**, 531-538, doi:10.1101/lm.1889510 (2010).

10 Zahm, D., Jensen, S., Williams, E. & Martin, J. r. Direct comparison of projections from the central amygdaloid region and nucleus accumbens shell. *Eur J Neurosci* **11**, 1119-1126 (1999).

11 Gerfen, C., Herkenham, M. & Thibault, J. The neostriatal mosaic: II. Patch- and matrix-directed mesostriatal dopaminergic and non-dopaminergic systems. *J Neurosci* **7**, 3915-3934 (1987).

12 Prensa, L. & Parent, A. The nigrostriatal pathway in the rat: A single-axon study of the relationship between dorsal and ventral tier nigral neurons and the striosome/matrix striatal compartments. *J Neurosci* **21**, 7247-7260 (2001).

13 Howland, J., Taepavarapruk, P. & Phillips, A. Glutamate receptor-dependent modulation of dopamine efflux in the nucleus accumbens by basolateral, but not central, nucleus of the amygdala in rats. *J Neurosci* **22**, 1137-1145 (2002).

14 Paxinos, G. & Watson, C. The rat in stereotaxic coordinates. *Academic Press, San Diego, CA* (1986).
